# Retraction Note: Role of the P2X7 receptor in inflammation-mediated changes in the osteogenesis of periodontal ligament stem cells

**DOI:** 10.1038/s41419-023-06390-y

**Published:** 2023-12-20

**Authors:** Xin-Yue Xu, Xiao-Tao He, Jia Wang, Xuan Li, Yu Xia, Yi-Zhou Tan, Fa-Ming Chen

**Affiliations:** State Key Laboratory of Military Stomatology, National Clinical Research Center for Oral Diseases and Shaanxi Engineering Research Center for Dental Materials and Advanced Manufacture, Department of Periodontology, School of Stomatology, Fourth Military Medical University, Xi’an, 710032 Shaanxi China

Retraction to: *Cell Death and Disease* 10.1038/s41419-018-1253-y, published online 08 January 2019

The authors have retracted this article because of problems with overlapping images in Figures 2, 4 and 7. All authors agree with this retraction.

